# Sirolimus as a second-line treatment for Graves’ orbitopathy

**DOI:** 10.1007/s40618-022-01862-y

**Published:** 2022-07-13

**Authors:** G. Lanzolla, M. N. Maglionico, S. Comi, F. Menconi, P. Piaggi, C. Posarelli, M. Figus, C. Marcocci, M. Marinò

**Affiliations:** 1 Endocrinology Unit I, Department of Clinical and Experimental Medicine, University of Pisa and University Hospital of Pisa, Via Paradisa 2, 56124 Pisa, Italy; 2 Present Address: Department of Orthopaedic Surgery, University of Pennsylvania, Philadelphia, PA USA; 3 Ophthalmopathy Unit I, Department of Surgical, Medical and Molecular Pathology, University of Pisa and University Hospital of Pisa, Via Paradisa 2, 56124 Pisa, Italy; 4 Department of Information Engineering, University of Pisa and University Hospital of Pisa, Via G. Caruso 16, 56122 Pisa, Italy

**Keywords:** Sirolimus, Graves’ disease, Graves’ orbitopathy, Rapamycin, Thyroid disease, Thyroid autoimmunity

## Abstract

**Objectives:**

A beneficial effect of sirolimus in Graves’ orbitopathy (GO) was reported, suggesting a possible use in clinical practice. We conducted an observational, single-centre, no-profit, clinical study to investigate the efficacy of sirolimus as a second-line treatment for moderate-to-severe, active GO compared with methylprednisolone.

**Methods:**

Data from consecutive patients given sirolimus (2 mg orally on first day, followed by 0.5 mg/day for 12 weeks) or methylprednisolone [500 mg iv/weekly (6 weeks), 250 mg/weekly (6 weeks)] as a second-line treatment were collected and compared. Primary objective: overall GO outcome at 24 weeks, based on a composite evaluation. Secondary objectives at 24 weeks: (1) improvement in quality of life, evaluated using a specific uestionnaire (GO-QoL); (2) reduction in proptosis; (3) reduction in the clinical activity score (CAS); (4) improvement of eye ductions; and (5) reduction in eyelid aperture.

**Results:**

Data from 30 patients (15 per group) treated between January 15, 2020, and June 15, 2021, were analysed. Proportion of GO responders (primary outcome) at 24 weeks was significantly greater in sirolimus group compared with methylprednisolone group (86.6% vs 26.6%; OR: 17.8; 95% CI from 2.7 to 116.8; *P* = 0.0026). GO-quality of life (GO-QoL) score was greater in sirolimus group. Proportion of proptosis responders was greater in sirolimus group, as well as proportion of clinical activity score (CAS) responders. No serious adverse events were observed, with no differences between groups.

**Conclusions:**

Sirolimus seems to be an effective second-line treatment for GO. Further randomized clinical trials are needed to confirm our observations.

## Introduction

Graves’ orbitopathy (GO) is the most common extrathyroidal manifestation of Graves’ disease (GD), due to autoantigens shared by orbital fibroblasts and thyroid epithelial cells [1, 2]. The recent acquisitions on GO pathogenesis promoted the ongoing changes in patient management, as well as the introduction of novel treatment procedures. High-dose intravenous glucocorticoids (ivGCs) are the most commonly used first-line treatment for moderate-to-severe, active GO, as recommended by the 2021 Guidelines of the European Group On Graves’ Orbitopathy (EUGOGO) [3]. Several options are currently available as second-line treatments, although their effectiveness and safety remain to be established with certainty [3, 4].

Sirolimus (rapamycin) is an immunosuppressive drug with anti-proliferative and anti-fibrotic properties, commonly used for prophylaxis of organ rejection in adult patients who have received kidney transplantation and have a mild to moderate immunological risk [5–7]. In addition, sirolimus has been approved for treatment of lymphangioleiomyomatosis and for medicated stents in patients undergoing coronary angioplasty [5–7]. The molecular target of sirolimus is a serine–threonine kinase (mammalian target of rapamycin, mTOR) that regulates cell growth, proliferation, motility, and survival [5]. Unlike other immunosuppressive drugs, sirolimus is not nephrotoxic and dosage adjustments are not necessary in patients with renal failure [5–7]. Furthermore, using relatively low doses, side effects are uncommon [5–7]. Two cases of patients with GO resistant to glucocorticoids and treated with sirolimus were described, with an apparent beneficial effect, suggesting a possible use of the drug in clinical practice [8, 9]. Based on these reports and in view of its mechanisms of action, we gave sirolimus off-label, as a second-line treatment, to patients with moderate-to-severe, active GO, in whom first-line treatment had failed and either there were contraindications to or the patients refused other second-line treatments (i.e. a second course of ivGCs). Here, we report a data analysis of these patients, compared with those of patients with moderate-to-severe, active GO treated with ivGCs, still as a second-line treatment, over the same period of time.

## Subjects and methods

### Study design

We performed an observational, single-centre, no-profit, clinical study to evaluate the effects of sirolimus as a second-line treatment on the outcome of moderate-to-severe, active GO compared to ivGCs. The investigation entailed data analysis of consecutive patients treated with either sirolimus or methylprednisolone over 18 consecutive months.

### Setting

The study was carried out at the University Hospital of Pisa, a tertiary referral Centre. The study was approved by the local Ethic Committee (Comitato Etico Area Vasta Nord-Ovest, approval no. 21672_MARINO) and performed in accordance with the International Conference on Harmonisation Good Clinical Practice guidelines and the principles of the Declaration of Helsinki. The study was registered in clinicaltrial.gov (registration no. NCT05345119).

### Participants

Data analysis was conducted in consecutive patients who fulfilled the following inclusion and evaded the following exclusion criteria.

Inclusion criteria: (1) men and women aged 18–75 years; (2) moderate-to-severe, active GO, defined as the presence of at least one of the following criteria associated with a clinical activity score (CAS) ≥ 3/7 points in most affected eye: (i) exophthalmos ≥ 2 mm compared with normal for gender and race; (ii) inconstant to constant diplopia; and (iii) lid retraction ≥ 2 mm; (3) previous treatment with ivGCs, performed more than 24 weeks before the current treatment; (4) written, signed informed consent including compliance with requirements and restrictions listed in the consent form.

Exclusion criteria: (1) optic neuropathy, as defined by the 2021 EUGOGO guidelines [3]; (2) treatment with glucocorticoids or other immunosuppressive medications, and/or selenium, and/or orbital radiotherapy and/or orbital decompressive surgery in the 24 weeks preceding the current treatment; (3) mental illness preventing comprehensive informed consent.

Signed informed consent was obtained from all patients.

### Outcomes

The primary endpoint of the study was the overall GO outcome at 24 weeks, based on a composite evaluation, as recommended by the 2021 EUGOGO guidelines [3, 10]. Patients were considered responders when at least two of the following criteria were fulfilled in the most affected eye, without worsening in any of the same measures in both eyes: (1) improvement in 5-point CAS (spontaneous and gaze-evoked pain excluded) by at least 1 point; (2) improvement in exophthalmos by at least 2 mm; (3) improvement in lid aperture by at least 2 mm; (4) improvement in eye muscle ductions ≥ 8°; (5) improvement in visual acuity by at least 0.2/1.

Secondary objectives were: (1) improvement in QoL (comparison between the two groups at 24 weeks); (2) reduction in proptosis at 24 weeks (percentage of subjects with a reduction ≥ 2 mm without worsening in the contralateral eye); (3) reduction in CAS at 24 weeks (percentage of subjects with a reduction in CAS by at least one point); (4) improvement of eye ductions at 24 weeks (percentage of subjects with increase in eye muscle ductions ≥ 8°); (5) improvement of diplopia at 24 weeks [percentage of subjects with disappearance or improvement (change from constant to inconstant, intermittent, or absent, from inconstant to intermittent or absent, or from intermittent to absent) of diplopia]; (6) reduction in lid aperture at 24 weeks (percentage of subjects with a reduction ≥ 2 mm); (7) TSH-receptor autoantibodies (TRAbs) at 24 weeks.

Safety objectives included adverse events documented and coded according to the standardized medical dictionary for regulatory affairs (MedDRA) [11], as recommended by the International Conference on Harmonisation of Technical Requirements for Registration of Pharmaceuticals for Human Use.

### Procedures

Starting at baseline, patients in the sirolimus group received a first dose of sirolimus of 2 mg orally on the first day, given approximately at 10 am, followed by 0.5 mg per day for 12 weeks. Patients in the methylprednisolone group received intravenous methylprednisolone according to the following, previously described [3], protocol: 500 mg/weekly (6 weeks), 250 mg/weekly (6 weeks) (cumulative dose 4.5 g). Patients in the methylprednisolone group were given omeprazole 20 mg/daily across the treatment period.

### Sources of data and measurements

An ophthalmological evaluation was performed at baseline and at 24 weeks, including: (1) exophthalmometry (Hertel exophthalmometer); (2) eyelid aperture; (3) assessment of diplopia (Gorman score) [3]; (4) ocular ductions; (5) corneal status; (6) fundi; (7) visual acuity (Snellen chart); and (8) CAS [12]. Patients were seen by two ophthalmologists (M.N.M, C.P) at the same time at all visits, in order to minimize inter- and intra-observer variations.

The following blood tests were performed at baseline, at 6, 12, 18, and 24 weeks: free thyroxine (FT4) and triiodothyronine (FT3), by chemiluminescence immunoassays (Vitros Immunodiagnostics, Raritan, NJ); thyroid stimulating hormone (TSH), by immunochemiluminometric assay (Immulite 2000, Siemens Healthcare, Gwynedd, UK). TRAbs were measured at baseline and at 24 weeks by enzyme-linked immunoassay (ElisaRSR™ TRAb 3rd Generation, Cardiff, UK). The following blood tests were performed at baseline and every two weeks up to 24 weeks: blood count, creatinine, AST, ALT, CPK, alkaline phosphatase, fasting blood glucose, total cholesterol, high-density lipoprotein-cholesterol, high-density lipoprotein-cholesterol and triglycerides, all by enzymatic-colorimetric assays (Roche, Mannheim, Germany). Rapamycin was measured in the sirolimus group at 12 weeks by chemiluminescent microparticle immunoassay (ARCHITECT Sirolimus, Abbott, Chicago, Illinois, USA).

Quality of life (QoL) was evaluated using a specific questionnaire for GO (GO-QoL) [13]. Patients filled the questionnaire at baseline and at 24 weeks. Questionnaire consists of two subscales: (1) visual functioning (eight questions concerning limitations attributable to decreased visual acuity, diplopia, or both), and (2) appearance (eight questions referring to limitations in psychosocial functioning attributable to changes in appearance). Questions are scored as severely limited (one point), a little limited (two points), or not limited at all (three points). The total score as well as the two subscales were converted into percentages according to the following formula: (total points × 100)/(number of questions answered × 3). A higher percentage means a better QoL.

Data were collected and recorded in a database. The following database validation procedures were employed: allowed character checks, batch totals, missing records check, cardinality check, digits check, consistency check, control totals, cross-system consistency check, data type check, hash totals, limit check, logic check, presence check, range check, spelling and grammar check, and uniqueness check.

### Bias

The study was not prospective neither randomized, and patients were offered treatment with sirolimus because of contraindications to methylprednisolone or if they refused methylprednisolone. In order to overcome the potential bias that could derive from lack of randomization, data analysis was conducted in patients who were not selected, but included by means of consecutive sampling. In addition, as reported below, the two study groups were similar for all features prior to treatment, which should guarantee a fair and correct comparison between the two treatment modalities.

### Study size

Being an observational, retrospective analysis, a sample size could not be calculated in advance. Instead, power calculations were conducted before data analysis in order to quantify the minimum detectable difference in the primary outcome measure. Given the size of each group (15 patients per group), the number of patients we analysed was estimated to be sufficient to achieve a 80% power in order to detect a difference > 45% via a two-sided *Z*-Test with unpooled variance, assuming that the percentage of responders in the control group (methylprednisolone) was 30%, as reported in a recent study in which the same regimen of methylprednisolone treatment was administered to patients for the same duration and a similar outcome measure was applied [14]. Accordingly, the study was powered to detect a percentage of responders in the group of patients treated with sirolimus greater than 75%.

### Statistical analyses

Continuous variables are presented as mean (SD) or median (IQR), as appropriate. Continuous variables were standardized to determine their effect on primary outcome.

Continuous variables were compared by ANOVA with Bonferroni’s correction or Mann–Whitney. Categorical data were compared by two-tailed Fisher’s exact test. Analyses were implemented using SPSS version 21.0 (IBM, New York, NY).

## Results

Between January 15, 2020, and June 15, 2021, we treated 15 patients with sirolimus, given as a second-line therapy for GO. The control, standard treatment group, comprised 15 consecutive patients treated with methylprednisolone over the same period, still as a second-line therapy. In all of these patients, the first-line treatment procedure (methylprednisolone in all cases) had failed, resulting in persistence of moderate-to-severe, active GO. In the sirolimus group, patients were given the medication off-label because of contraindications to ivGC (gastritis in 2 patients, severe liver steatosis in 3 patients, severe osteoporosis in 2 patients), or because they refused to undergo a second course of methylprednisolone (8 patients).

Demographic and clinical variables of the two groups at baseline are shown in Table 1, none of which differed significantly. All patients were on levothyroxine treatment in both groups, having been previously treated with radioiodine or thyroidectomy, in all cases more than 24 weeks before the baseline observation. All patients were euthyroid, with FT4 and TSH within normal range and no differences between groups. As expected from patients undergoing a second-line treatment, the duration of GO was relatively long. All patients had a moderate-to-severe, active GO, according to the criteria proposed by EUGOGO [3], thereby prompting a treatment. All patients had previously received a course of methylprednisolone (total dose 4.5 g) more than 24 weeks before the baseline observation.

**Table 1:** Demographic and clinical data at baseline

Feature	Sirolimus	Methylprednisolone	Statistics
Gender	Men: 1 (6.6); Women: 14 (93.3)	Men: 2 (13.3); Women: 13 (86.6)	OR: 0.46 95% CI from 0.037 to 5.74 *P* = 0.55
Age (yr.)	55.6 (9.9)	52.4 (11.2)	Mean difference: − 1.5 95% CI from − 8 to 5 *P* = 0.63
Smoking habits (no.)	Nonsmokers: 4 (26.6) Ex-smokers: 7 (46.6) Current smokers: 4 (26.6)	Nonsmokers: 5 (33.3) Ex-smokers: 6 (40) Current smokers: 4 (26.6)	Pearson’s Chi^2^: 0.18 *P* = 0.91
Thyroid Treatment	l-thyroxine: 11 (100) [10 (66.6) after radioiodine, 5 (33.3) after thyroidectomy]	l-thyroxine: 15 (100) [11 (73.3) after radioiodine, 4 (26.6) after thyroidectomy]	N/A
Previous treatment/s for Graves’ orbitopathy	Methylprednisolone: 15 (100)	Methylprednisolone: 15 (100)	N/A
FT4 (ng/dL; reference range: 0.7–1.7)	1.2 (0.2)	1.3 (0.4)	Mann Whitney *U*: 89.5 *P* = 0.35
TSH (mU/L; reference range: 0.4–4)	1.4 (0.6–2.8)	0.6 (0.2–2.3)	Mann Whitney *U*: 97 *P* = 0.53
TRAbs (IU/L; cut-off < 1.5)	5.3 (2.7–35.1)	14.8 (2.8–62.2)	Mann Whitney *U*: 103 *P* = 0.71
Graves’ orbitopathy duration (mo.)	36 (30.5–66)	27 (18–48)	Mann Whitney *U*: 81 *P* = 0.2082
Exophthalmometry (most affected eye) (mm)	23.4 (3.5)	23.2 (2.4)	Mean difference: 0.2 95% CI from − 2 to 2.4 P = 0.85
Clinical Activity Score (points)	4.6 (1.2)	3.8 (1.2)	Mean difference: 0.6 95% CI from − 0.3 to 1.3 P = 0.19
Eyelid aperture (mm)	12.6 (2.3)	13.6 (3)	Mean difference: − 1 95% CI from − 3 to 1 *P* = 0.32
Sum of eye ductions (degrees)	270 (41.4)	253.6 (52.3)	Mann Whitney *U*: 90.5 *P* = 0.37
Diplopia (Gorman’s score)	Absent: 4 (26.6) Intermittent: 2 (13.3) Inconstant: 6 (40) Constant: 3 (20)	Absent: 2 (13.3) Intermittent: 1 (6.6) Inconstant: 11 (73.3) Constant: 1 (6.6)	Pearson’s Chi^2^: 3.47 *P* = 0.32
Best corrected visual acuity (most affected eye) (decimals)	1 (0)	1 (0)	Mean difference: 0 95% CI from 0 to 0 *P* = 1
Quality of life total score (%)	63.7 (13.9)	61.8 (9.7)	Mean difference: 1.8 95% CI from − 6.8 to 10.8 *P* = 0.65
Quality of life functioning subscale (%)	62.5 (15.4)	65.8 (17.9)	Mean difference: − 3.3 95% CI from − 16.2 to 8.7 *P* = 0.56
Quality of life appearance subscale (%)	67.5 (14.1)	58.7 (9.5)	Mean difference: 4.1 95% CI from − 0.4 to 17.5 *P* = 0.064

Data are mean (SD), median (IQR) or *n* (%)

*FT4* free thyroxine, *TSH* thyroid stimulating hormone, *TRAbs* thyroid stimulating hormone receptor autoantibodies

In the sirolimus group, serum rapamycin was measured at the end of treatment, namely at 12 weeks, resulting in a median concentration of 2.3 ng/ml (IQR: 1.9–2.5).

**Fig. 1:**
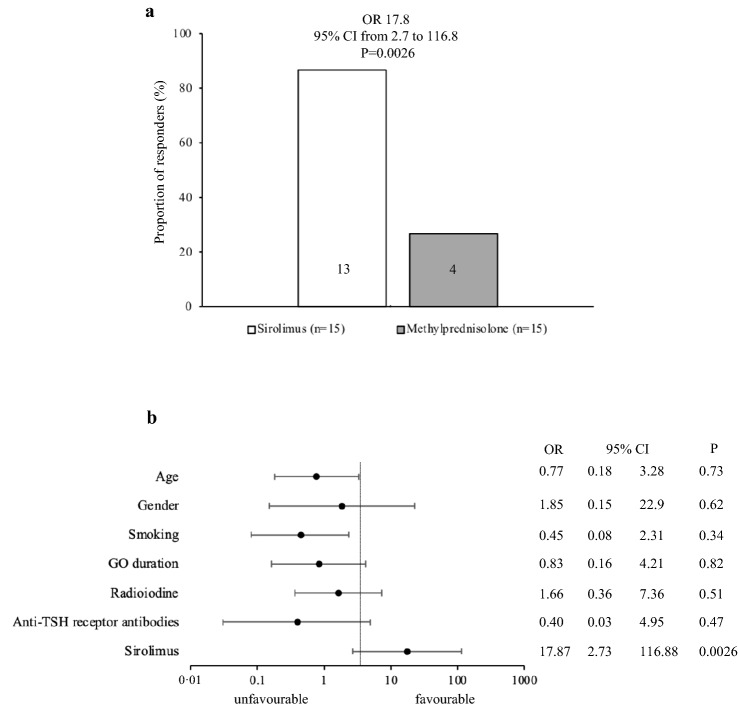
Primary endpoint.** a** Overall outcome of Graves’ orbitopathy; **b** effect of various determinants on the overall outcome of Graves’ orbitopathy

As shown in Table 2 and Fig. 1a, using a composite evaluation, the proportion of GO responders at 24 weeks (primary endpoint) was significantly greater in the sirolimus group compared with the methylprednisolone group. Age, gender, smoking, radioiodine, TRAbs and GO duration did not affect the overall GO outcome (Fig. 1b), nor they did affect secondary endpoints (not shown). The only variable affecting significantly the primary endpoint was sirolimus (Fig. 1b).

**Table 2:** Primary and secondary study endpoints at 24 weeks

Outcome	Sirolimus	Methylprednisolone	Statistics
Overall response	13/15 (86.6)	4/15 (26.6)	OR: 17.8 95% CI from 2.7 to 16.8 *P* = 0.0026
Quality of life total score (%)	77.5 (13.7)	65.8 (8.5)	Mean difference: 11.4 95% CI from 2.9 to 20 *P* = 0.010
Quality of life functioning subscale (%)	86.2 (29.5)	63.3 (11.2)	Mean difference 22.5 95% CI from 5.8 to 39.5 *P* = 0.010
Quality of life appearance subscale (%)	77.9 (19.1)	72 (17.5)	Mean difference: 5.8 95% CI from 7.9 to 19.5 *P* = 0.39
Outcome of exophthalmos (responders)	12/15 (80)	2/15 (13.3)	OR: 26 95% CI from 3.6 to 183.4 *P* = 0.0011
Outcome of clinical activity score (responders)	13/15 (86.6)	5/15 (33.3)	OR: 13 95% CI from 2 to 81.4 *P* = 0.0062
Outcome of eye ductions	3/15 (20)	4/15 (26.6)	OR 0.6 95% CI from 0.1 to 3.7 *P* = 0.66
Outcome of diplopia (only patients with diplopia at baseline)	7/11 (63.6)	3/13 (23)	OR: 5.8 95% CI from 0.9 to 34.6 *P* = 0.052
Outcome of eyelid aperture	4/15 (26.6)	3/15 (20)	OR: 1.4 95% CI from 0.2 to 8 *P* = 0.66
Outcome of visual acuity	N/A	N/A	N/A
Thyroid stimulating hormone receptor autoantibodies (IU/L; cut-off < 1.5)	4.2 (1–29.5)	24.7 (7.2–151.5)	Mann–Whitney *U*: 18 *P* = 0.18

Data are *n* (%), mean (SD) or median (IQR)

**Fig. 2:**
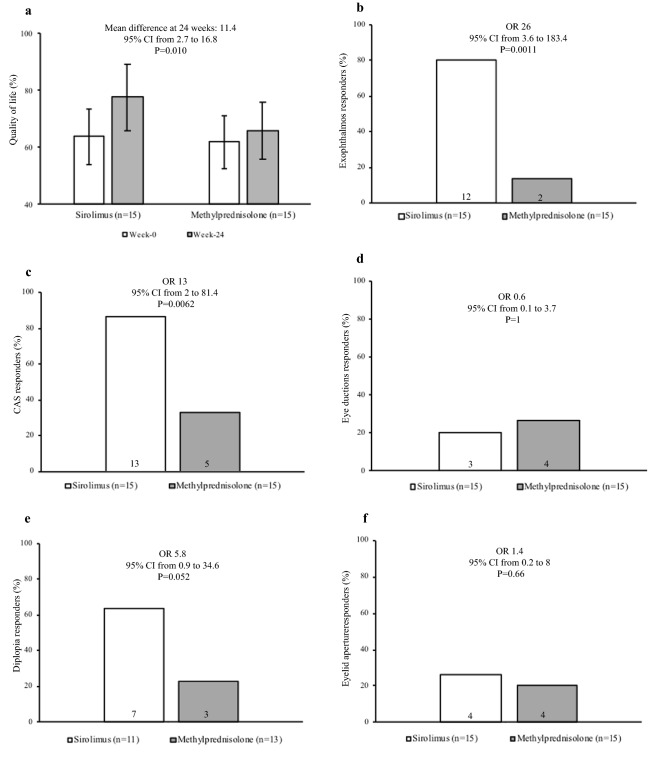
Secondary endpoints.** a** Quality of life; **b** proptosis; **c** clinical activity score (CAS); **d** eye ductions; **e** diplopia; **f** eyelid aperture

The total GO-QoL score at 24 weeks (secondary endpoint) was significantly greater in the sirolimus group (Table 2, Fig. 2a). Concerning the two subscales of GO-QoL, the functioning subscale was greater in the sirolimus group, whereas the appearance subscale did not differ statistically between the two groups (Table 2).

Still as secondary endpoints, we evaluated the outcome of the single eye features and the behaviour of TRAbs. As shown in Table 2, the proportion of proptosis and CAS responders was significantly greater in the sirolimus group (Table 2, Fig. 2b, c), whereas eye ductions and eyelid width responders did not differ between the two groups (Table 2, Fig. 2d, f). However, there was a trend to a greater proportion of diplopia responders in the sirolimus group, although the difference did not reach statistical significance (Table 2, Fig. 2e). TRAbs levels at 24 weeks did not differ between the two groups (Table 2). Baseline and 24-week values of QoL, proptosis, CAS, eye ductions, eyelid aperture, visual acuity and TRAbs are reported in Table 3.

**Table 3:** Eye features and quality of life at baseline and at 24 weeks

Feature	Sirolimus	Methylprednisolone
Quality of life (%)	Baseline: 63.7 (13.9) 24 weeks: 77.5 (13.7)	Baseline: 61.8 (9.7) 24 weeks: 65.8 (8.5)
Quality of life functioning subscale (%)	Baseline: 62.5 (15.4) 24 weeks: 86.2 (29.5)	Baseline: 65.8 (17.9) 24 weeks: 63.3 (11.2)
Quality of life appearance subscale (%)	Baseline: 67.5 (14.1) 24 weeks: 77.9 (19.1)	Baseline: 58.7 (9.5) 24 weeks: 72 (17.5)
Exophthalmometry (most affected eye) (mm)	Baseline: 23.4 (3.5) 24 weeks: 21.2 (3.5)	Baseline: 23.2 (2.4) 24 weeks: 23 (2.7)
Clinical Activity Score (points)	Baseline: 4.6 (1.2) 24 weeks: 2.2 (1.1)	Baseline: 3.8 (1.2) 24 weeks: 2.8 (0.9)
Eyelid aperture (mm)	Baseline: 12.6 (2.3) 24 weeks: 11.2 (2.3)	Baseline: 13.6 (3) 24 weeks: 13.4 (3.1)
Sum of eye ductions (degrees)	Baseline: 270 (41.4) 24 weeks: 271 (42.4)	Baseline: 253.6 (52.3) 24 weeks: 254 (60.6)
Best corrected visual acuity (most affected eye) (decimals)	Baseline: 1 (0) 24 weeks: 1 (0)	Baseline: 1 (0) 24 weeks: 1 (0)
TRAbs (IU/L; cut-off < 1.5)	Baseline: 5.3 (2.7–35.1) 24 weeks: 4.2 (1–29.5)	Baseline: 14.8 (2.8–62.2) 24 weeks: 14.1 (2.3)

Data are mean (SD) or median (IQR)

*TRAbs* thyroid stimulating hormone receptor autoantibodies

Eight adverse events occurred in 8 patients (26.6%), with no serious and/or unexpected events and no difference between groups (Table 4). None of the patients in both groups discontinued the medications or required dose reductions.

**Table 4:** Adverse events

	Sirolimus	Methylprednisolone
Total	4/15 (26.6)	4/15 (26.6)
Cardiac disorders	0 (0)	1 (6.6)
Palpitations	0 (0)	1 (6.6)
Infections	2 (13.3)	2 (13.3)
Cystitis	2 (13.3)	2 (13.3)
Metabolism and nutrition disorders	2 (13.3)	1 (6.6)
Hyperglycaemia	1 (6.6)	1 (6.6)
Hypercholesterolemia	1 (6.6)	0 (0)

Data are *n* (%)

## Discussion

The response rate of moderate-to-severe, active GO to methylprednisolone is known to be rather variable, ranging from ~ 25 to ~ 90% depending on several factors, including GO duration, previous treatments, methylprednisolone dosage, criteria to define GO outcome, smoking habits and others still to be defined [14–16]. In addition, steroids carry several contraindications that may prevent their use and can be associated with adverse events leading to dose reduction or withdrawal of the drugs [14, 15]. The ongoing acquisitions on GO pathogenesis have driven, over the years, several changes in management of patients, specially through the introduction of novel treatment procedures, namely drugs to be added to methylprednisolone [17], or alternative medications to be given when steroids are not effective [3]. In this regard, the second-line treatments for GO recommended by the 2021 EUGOGO guidelines include a second course of ivGCs as monotherapy or associated with radiotherapy, steroid-sparing agents (cyclosporine or azathioprine) associated with oral prednisone, or other immunosuppressive agents such as rituximab, teprotumumab, tocilizumab, all in monotherapy [3]. However, the response rate to these treatments is unclear, except for teprotumumab, which, unfortunately, is extremely costly and not available worldwide [4].

The present, observational investigation was aimed at evaluating the effects of sirolimus as a second-line treatment in patients with moderate-to-severe GO in whom a course of intravenous methylprednisolone had failed. As mentioned above, sirolimus is an immunosuppressive agent with anti-proliferative and anti-fibrotic properties, due to targeting of mTOR [5, 6]. In spite of its efficacy in inhibiting both innate and adaptive immune responses, sirolimus has limited side effects compared with immunosuppressive drugs with similar properties and mechanisms of action, in particular cyclosporine [5–7]. Thus, unlike cyclosporine, sirolimus is not nephrotoxic and dose adjustments are not required in patients with renal failure. Furthermore, using low dosages, side-effects are uncommon [5–7]. The only contraindications to sirolimus are hypersensitivity to the active substance or to any of the excipients, age < 18 yr., and pregnancy [5]. In 2007 and 2019, two cases of patients with GO resistant to glucocorticoids and treated with sirolimus were reported, with an apparent beneficial effect [8, 9]. Based on these reports and in view of the mechanisms of action of sirolimus, beginning in 2020, we started giving sirolimus off-label, as a second-line treatment, to patients with moderate-to-severe, active GO in whom a previous course of methylprednisolone had failed. Here, we reported the findings obtained in the first 15 consecutive patients treated with sirolimus, who were compared retrospectively with those of 15 consecutive patients treated with methylprednisolone, still as a second-line therapy, over the same period of time. Our results can be summarized as follows.

The overall response of GO at 24 weeks (primary endpoint) was remarkably greater in patients given sirolimus compared with those given methylprednisolone, being GO responders in the sirolimus group 86.6% vs 26.6% in the methylprednisolone group. This was associated with a better QoL and a greater proportion of proptosis (80% vs 13.3%) and CAS (86.6% vs 33.3%) responders (all secondary endpoints). Although the proportion of eye duction responders did not differ between the two groups, there was a trend to a better outcome of diplopia in the sirolimus group. No major adverse events related to sirolimus were observed, and no patients required dose reduction or discontinued the medication.

To our knowledge, this is the first report on the use of sirolimus in a series of patients with GO. The effects we observed can be explained by the mechanisms of action of the drug. In addition to blocking T-cell activation by inhibiting the calcium-dependent and calcium-independent translation signals, sirolimus binds to the FKPB12 protein to form a complex that leads to inhibition of mTORC1, which is essential for the activity of CD4- and CD8-positive cells, both involved in GO, as well as for adipogenesis [6, 7, 18, 19]. In vitro studies have shown that sirolimus-mediated inhibition of mTORC1 is followed by a block in adipogenesis in preadipocytes and fibroblasts from GO patients. In addition, by blocking the FRAP/mTOR signaling pathway, sirolimus reduces the synthesis of IL-16 promoted by circulating IgGs in orbital fibroblasts [18–20]. IL-16, together with the chemokine RANTES, represents one of the main mediators of "trafficking" of CD4-positive T lymphocytes in GO [21, 22]. Moreover, circulating IgGs that stimulate IL-16 production in orbital fibroblasts likely act via the signaling pathway of the insulin-like growth factor 1 (IGF-1), and teprotumumab, a monoclonal anti-IGF-1 receptor (IGF-1R) antibody, inhibits the process by effectively reducing IL-16 production and limiting CD4-positive T lymphocyte trafficking [4]. These effects have a clinical counterpart in that teprotumumab has been shown to be very effective for GO [4]. Given the molecular mechanisms of action of sirolimus, as teprotumumab, the drug may block IGF-1 signaling as well as other pathways involved in migration, proliferation, differentiation and cell activation, resulting in similar clinical effects, as we observed here. Furthermore, recent in vitro studies have shown that sirolimus affects tissue remodelling also by negatively regulating fibroblast migration and fibroblast transition into myofibroblasts, therefore reducing the production of collagen and extracellular matrix [20, 23]. The anti-fibrotic effect of sirolimus has also been demonstrated in vivo in a murine model [24–26], and it has been proposed as one of the possible explanations for the efficacy of this drug in the two patients with GO reported previously [8, 9]. Overall, sirolimus blocks T cells, limits the production of proinflammatory cytokines and has a negative role both on adipogenesis and differentiation of fibroblasts into myofibroblasts, suggesting that it might have an important role on several elements involved in the pathogenesis of GO, which explains our findings.

The major limitation of the present study is its observational nature, to overcome which we are currently planning a Phase II, randomized, clinical trial (SIRGO, clinicaltrial.gov no. NCT04598815). In spite of this, the remarkable difference in terms of GO outcome between patients treated with sirolimus and those treated with methylprednisolone over the same period of time and starting from the same conditions is somehow reassuring on the effectiveness of sirolimus, and certainly quite promising. Interestingly, one of the GO features that responded better to sirolimus was proptosis, an observation similar to that obtained with teprotumumab [4], in line with the possibility that the two drugs act on a common pathway. As mentioned above, sirolimus was used as a second-line treatment, but it may be effective also as a first-line treatment, which will be investigated in the clinical trial mentioned above.

Given the central role of TSH-R in the pathogenesis of GO [27], a reduction in TRAbs would have been expected in patients given sirolimus. However, this assumption is not necessarily straightforward, considering that sirolimus is a specific inhibitor of mTOR, which is listed among the players involved in orbital fibroblasts proliferation and fibrosis. Thus, it may well be that the beneficial effects of sirolimus reflect its anti-proliferative and anti-fibrotic actions in orbital fibroblasts, namely downstream the immune system activation against TSH-R, and therefore, regardless of the serum concentrations of TRAbs.

The median concentration of rapamycin in our patients, measured at the end of treatment, was 2.3 ng/ml, which is about half the desired concentrations in patients given sirolimus for lymphangioleiomyomatosis and for prevention of organ rejection after kidney transplantation. This reflects the low dosage of the medication we gave to patients, which we choose arbitrarily in view of the fact that the immunosuppressive and antifibrotic effects required for GO should be considerably lesser than the ones required for the two conditions mentioned above. Thus, as a matter of fact, the dose used here proved to be effective in ameliorating GO.

Sirolimus is known to act on bone turnover, which was not investigated here and will certainly be investigated in our upcoming studies.

In conclusion, sirolimus is a novel, promising medication for GO. At the dosage used, sirolimus appears to be quite safe with no major adverse events, although this awaits confirmation in a larger series of patients. In addition, sirolimus is quite affordable. Thus, its overall cost per patient at the dosage used here ranges between 270 and 290 Euros, which also makes it a suitable candidate for GO treatment.
